# Notes and News

**Published:** 1895-10-05

**Authors:** 


### Notes and News.

### THE INTRODUCTORY ADDRESSES AT THE MEDICAL SCHOOLS.

### St. George's Hospital.

Mr. George Pollock, F.R.C.S., after a short sketch of the history of the hospital and the school, insisted on the importance, throughout the medical man's career, of sympathy, kindness, and consideration in dealing with patients of whatever degree. He then went on to draw attention to the desirability of studying the idiosyncrasies of patients, and of treating these peculiarities with respect, not ridicule. As illustrating this, he mentioned the case of a person who could not eat rice without producing alarming symptoms. It seemed almost impossible to believe that so innocent a vegetable substance, on which thousands of natives of India and China almost entirely subsist, should possess such deleterious properties; and to test the matter some friends of the person referred to, knowing that he was fond of biscuits, had some prepared with one grain of rice in each. These biscuits were placed near him after dinner, and he partook of two or three of them. He became uncomfortable, and had to leave the table, observing at the same time that, if he were not morally certain that he had not partaken of rice at dinner, he was being poisoned by it. But the same thing applied to many drugs; "therefore," said Mr. Pollock, "be careful in prescribing whenever your patient may tell you that a certain drug does not agree with him." Many illustrations were given of the importance of this injunction.

### Middlesex Hospital.

The address was delivered by Dr. W. Julius Mickle, F.R.C.P., who, taking the manner in which a child gains knowledge of a simple object as the type of the methods by which knowledge should be attained, showed how medical study should be arranged to the best advantage. It was pointed out that in order to be natural and fitting in method the process must be gradual; that it must begin with the most simple, which must be thoroughly mastered before the more complex is undertaken, and that all the senses should co-operate in the process. All the activities of the mind should be employed in co-operation with one another; wide culture was of the greatest importance in the student of medicine, and as showing the advantage of a broad knowledge of many sciences the names of Hulke and Bristowe were held up as examples.

### Westminster Hospital.

The introductory lecture was given by Dr. S. Monckton Copeman, who, after paying a feeling tribute to the memory of the late Dr. Octavius Sturges, passed on to a review of some of the more important developments of modern medicine, especially in relation to the treatment of disease. In this connection attention was directed more particularly to the employment of preparations of an organic nature, often in the form of extracts of certain organs, in diseases in which those particular organs were affected, and to the bearing of bacteriological research on the diagnosis and treatment of specific infectious maladies.

The lecturer concluded with a short account of Dr. Copeman's own work in connection with small-pox and vaccinia, which had resulted in the cultivation of a specific organism common to both these diseases, calves having been successfully inoculated with the cultures so produced. The hope, therefore, was expressed that we may in the future, in addition to the protection afforded by vaccination, have a means of combating the inroads of small-pox when the system has actually been invaded by the disease.

### Guy's Hospital.

Dr. George De Ath read a paper before the Guy's Physical Society, in which he warned his hearers against the growing tendency which existed to render medical topics popular. The profession, he said, attempted to boil down the hard bones of science to suit the digestion of the public intellect, and flooded the Press with adulterated solutions of knowlege. This was called the popularisation of knowledge, but it was in reality the prostitution of science.

### University College.

Professor J. Rose Bradford, F.R.S., delivered the address at University College, discussing more especially the position that the preliminary sciences —biology, anatomy, and physiology—should occupy in the medical curriculum. The burden laid on the student of medicine was so great and increasing that it was essential that too much time should not be spent in studies taking him away from the hospital ward. No amount of biological—or, for the matter of that, of pathological and bacteriological—knowledge would make the capable physician or surgeon; that was only to be acquired in the hospital wards.

### St. Mary's Hospital.

Dr. P. A. Laurie took for his subject "The Medical Profession and Unhealthy Trades," and after pointing out the wide ramifications of existing factory legislation, and the heavy responsibilities and complex duties with reference to the health of factory workers which had been placed upon the Home Office, stated that these duties could not be satisfactorily fulfilled by the ordinary staff of inspectors. Expert and special medical knowledge was essential.

